# Seek First to Understand: Sex-Specific Outcomes of Patients With Calcified Coronary Arteries Treated With Intravascular Lithotripsy

**DOI:** 10.1016/j.jscai.2023.101120

**Published:** 2023-11-27

**Authors:** Kathryn I. Sunthankar, Nadia R. Sutton

**Affiliations:** a Division of Cardiovascular Medicine, Department of Medicine, Vanderbilt University Medical Center, Nashville, Tennessee; b Department of Biomedical Engineering, Vanderbilt University, Nashville, Tennessee

**Keywords:** coronary calcification, intravascular lithotripsy, percutaneous coronary intervention, sex-specific outcomes

The presence and extent of coronary calcification has a tremendous impact on the success of percutaneous coronary intervention (PCI). Technologies for calcium modification have evolved to include rotational atherectomy; orbital atherectomy; laser; specialty balloons, such as cutting, scoring, and high-pressure balloons; and more recently, intravascular lithotripsy (IVL). PCI, on calcified vessels requiring atherectomy, is associated with longer procedural times, slow or no reflow, and coronary perforation.^1^ Intraprocedural complications, such as coronary dissection, cardiac tamponade, and significant bleeding, have been observed to occur at a higher rate in women compared with men in some studies but not others.^2^, ^3^, ^4^

The Disrupt CAD I-IV studies were single-arm studies of the safety and success of IVL, noting few serious procedural complications, such as dissection, perforation, or slow flow.^5^^,^^6^ These favorable clinical outcomes and ease of use have led to increasing use of IVL. Subgroup analyses have the potential to identify populations that could benefit or be harmed, and detailed, sex-specific outcomes for patients undergoing IVL have not been previously reported. Seeking to fill this gap in knowledge, in this issue of *JSCAI*, Frampton et al^7^ contribute an important analysis of sex-specific outcomes for patients with heavily calcified coronary arteries undergoing PCI treated with IVL. The study includes patients from the Disrupt CAD III and IV studies, totaling 448 individuals and including 106 women (24%). Sex-specific groups were assessed for the primary end point of major adverse cardiac events (MACE), which represented a composite of cardiac death, all myocardial infarction, or target vessel revascularization. Additional outcomes included target lesion failure and angiographic outcomes, including acute gain, minimum lumen diameter, and residual diameter stenosis.

There was no difference in rates of MACE between men and women after 30 days and 1 year. Interestingly, despite having a similar extent of lesion calcium compared with men, women required fewer and shorter stents as well as had shorter procedure time and required fewer lithotripsy catheters and pulses to achieve appropriate lesion modification. There were no serious angiographic adverse outcomes in women and only a few in men. Although sex was not an independent predictor of MACE at 1 year, longer lesions and bifurcation lesions were independent predictors of MACE and target lesion failure at 1 year. The proportion of individuals with intravascular imaging is not reported in the current study; it is noted that women were more likely than men to have a procedure with femoral access. This was probably due to a higher proportion of women with radial arteries too small to be utilized. Vascular access complications were not a prespecified end point in the original Disrupt CAD III or IV studies and are, therefore, not reported. Although IVL has demonstrated similar safety in men and women, it remains important to continue to optimize other procedural aspects to improve clinical outcomes for patients.

Currently, we do not have data from randomized studies that directly compare modalities most harnessed for the treatment of heavily calcified coronary lesions: IVL, rotational atherectomy, and orbital atherectomy. Given the current climate for funding of research studies, such a trial is unlikely to transpire. Even if such a trial would be constructed, it would be subject to the same limitations as other randomized studies, namely, the willingness of operators to enroll their patients into a randomized study when the treatment patterns and a sense of patient safety and risk are already established. Observational (real-world) studies are biased by patient selection for specific therapies, operator preference, institutional practices, and risk tolerance. These limitations are not specific to the study of treating calcified coronary artery lesions. Studies such as this subanalysis, which stemmed from a single-arm study, are likely the most solid evidence that the field will be able to acquire to address questions about therapies, such as IVL, to treat calcified coronary arteries. Increasingly, operators opt for an “IVL first” approach. One drawback of this trend is the potential for declining familiarity with atherectomy devices, which were already previously used in a minority of cases and which sometimes are the only option for modifying calcium. Utilization of atherectomy in only the highest-risk patients could result in further declining use and risk aversion. Therefore, as a field, it is important to continue to point out the necessity of complementary devices and to continue to use intravascular imaging to choose the correct tool for the correct patient.

The current study adds to our understanding by demonstrating that IVL relays similar benefits to men and women. This observation is not taken for granted, given the known increased risks of PCI in calcified coronary arteries, particularly in older, frail women. Future efforts, such as the Equity in Modifying Plaque of Women with Undertreated Calcified Coronary Artery Disease (EMPOWER CAD),^8^ which seeks to enroll 400 women referred for PCI with coronary IVL, are focused on breaking down sex-based disparities in the representation of women in clinical studies. EMPOWER CAD will further expand the knowledge base of the outcomes of women treated with PCI and IVL. Overall, the current study provides a positive outlook for women undergoing complex PCI for calcified coronary lesions requiring IVL. It is another strong step toward understanding how to improve outcomes and reduce risk^7^^,^^9^ for a population that, historically, has been understudied.
